# Cryptococcal Virulence in Humans: Learning From Translational Studies With Clinical Isolates

**DOI:** 10.3389/fcimb.2021.657502

**Published:** 2021-04-21

**Authors:** Herdson Renney de Sousa, Stefânia de Frazão, Getúlio Pereira de Oliveira Júnior, Patrícia Albuquerque, André Moraes Nicola

**Affiliations:** ^1^Microbiology, Immunology and Biotechnology Laboratory, Faculty of Medicine, University of Brasília, Brasília, Brazil; ^2^Laboratory of Molecular Biology of Pathogenic Fungi, Department of Cell Biology, Institute of Biological Sciences, University of Brasília, Brasília, Brazil; ^3^Division of Allergy and Inflammation, Department of Medicine, Beth Israel Deaconess Medical Center, Harvard Medical School, Boston, MA, United States; ^4^Faculty of Ceilândia, University of Brasília, Brasília, Brazil; ^5^Graduate Program in Genomic Sciences and Biotechnology, Catholic University of Brasília, Brasília, Brazil

**Keywords:** cryptococcosis, *Cryptococcus neoformans*, *Cryptococcus gattii*, meningitis, virulence

## Abstract

Cryptococcosis, an invasive mycosis caused by *Cryptococcus* spp, kills between 20% and 70% of the patients who develop it. There are no vaccines for prevention, and treatment is based on a limited number of antifungals. Studying fungal virulence and how the host responds to infection could lead to new therapies, improving outcomes for patients. The biggest challenge, however, is that experimental cryptococcosis models do not completely recapitulate human disease, while human experiments are limited due to ethical reasons. To overcome this challenge, one of the approaches used by researchers and clinicians is to: 1) collect cryptococcal clinical isolates and associated patient data; 2) study the set of isolates in the laboratory (virulence and host-pathogen interaction variables, molecular markers); 3) correlate the laboratory and patient data to understand the roles fungal attributes play in the human disease. Here we review studies that have shed light on the cryptococcosis pathophysiology using these approaches, with a special focus on human disease. Isolates that more effectively evade macrophage responses, that secrete more laccase, melanize faster and have larger capsules in the cerebrospinal fluid are associated with poorer patient outcomes. Additionally, molecular studies have also shown that cryptococcal clades vary in virulence, with clinical impact. Limitations of those studies include the use of a small number of isolates or retrospectively collected clinical data. The fact that they resulted in very important information is a reflection of the impact this strategy has in understanding cryptococcosis and calls for international collaboration that could boost our knowledge.

## Introduction

The genus *Cryptococcus* is one of the deadliest among those that cause systemic mycoses in humans (Bongomin et al., 2017). Pathogenic species are part of the *C. neoformans* and *C. gattii* complexes, but the genus includes many other species that are rarely or never pathogenic to humans and animals (Enoch et al., 2017; Kwon-Chung et al., 2017). These encapsulated yeasts are normally found in the soil and trees. How these environmental microbes have evolved complex virulence factors that allow them to survive and multiply in the human host is an important question that has been the target of research for decades. Beyond being interesting from an evolutionary point of view, cryptococcal virulence and its interaction with the human host are also clinically critical.

A global epidemiology study estimated that each year the disease kills 181,100 out of 223,100 individuals it affects (Rajasingham et al., 2017). Mortality rate estimates in this study were of up to 70% in low-income countries; however, even in optimal settings in high-income countries in North America and Europe, the estimated mortality rates range around 20% - 30%. One of the reasons for that is the limited arsenal of antifungals to treat the disease, with basically just three drugs: amphotericin B, flucytosine and fluconazole. Amphotericin B can be cheap (US$ 3.80 per dose) but very toxic in its deoxycholate formulation (Bicanic et al., 2005), whereas lipidic formulations are safer (Faustino and Pinheiro, 2020) but very expensive (US$ 443 per dose for the liposomal and US$ 506 per dose for the lipid complex formulations, all prices in Brazil for the year 2016) (Borba et al., 2018). Flucytosine improves the efficacy of amphotericin therapy, but is toxic and not available in most countries, whereas fluconazole is ineffective as monotherapy (Lu et al., 2019). As exemplified by tetanus, diphtheria and several other infectious diseases, virulence factors are frequent therapeutic and prophylactic targets. Studying the host response is also very important, because it could lead to host-directed therapies that would be very important in a disease in which immunocompromise is the major risk factor. Thus, research on virulence and host-pathogen interaction has great potential in decreasing the disease burden of cryptococcosis.

An important problem, however, is that it is very difficult to study virulence and host-pathogen interaction in human beings. A key experiment in determining a microbe’s virulence attributes is to impair functionality of the genes that encode them and compare the outcomes of experimental infection with the original strain and the mutants. On the host side, a frequent approach to test hypotheses involves infecting genetically modified animals and comparing the outcome with wild-type animals. However, neither approach is feasible in humans. Experiments like these have been done extensively in animal models, but they do not always recapitulate human diseases. For example, mice succumb to cryptococcal infection (Goldman et al., 1996; Carroll et al., 2007) and commonly used immortalized macrophages have weak *in vitro* fungicidal activity (Pan et al., 2009). In contrast, an intact immune response in healthy human beings effectively deals with the fungus in most individuals as evidenced by the fact that most people are infected with *Cryptococcus* spp. during their early life (Goldman et al., 2001) but the incidence of cryptococcosis in the HIV uninfected is a few cases per million people per year (Chen et al., 2000; Mirza et al., 2003).

**Figure 1:**
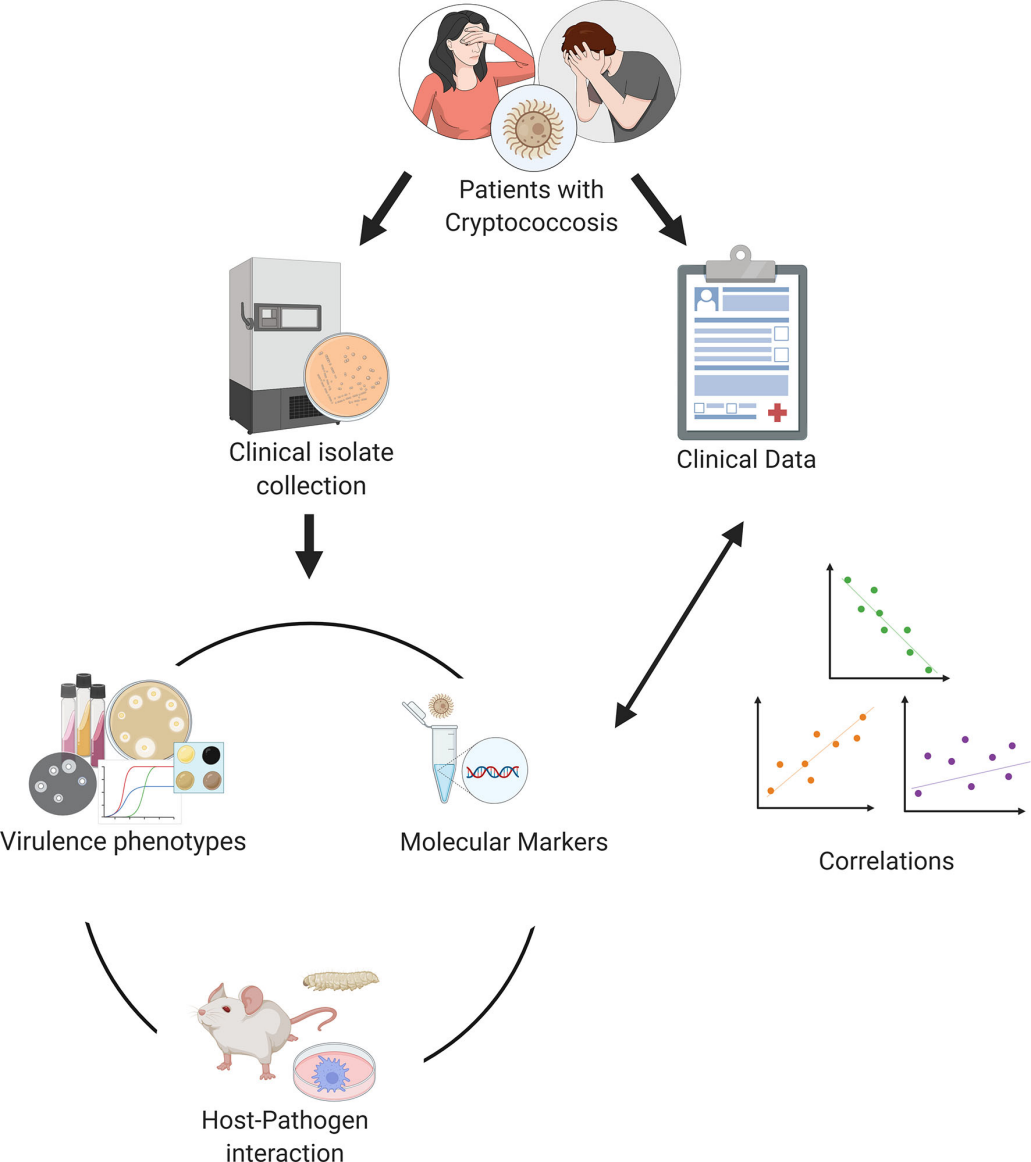
Experimental strategy of translational studies in cryptococcosis. Individual research groups collect patient data and clinical isolates. Information about the isolates is then collected experimentally in the laboratory and finally correlated with clinical data. Figure generated using Biorender.com.

One strategy that researchers have used to obtain information about virulence and host-pathogen interaction in humans starts with collecting clinical isolates together with information from the patients from whom each isolate was obtained. The microorganisms are then studied in the laboratory, where virulence attributes and host-pathogen interaction variables are systematically measured. The laboratory measurements are then correlated with patient data, revealing how virulence attributes are associated with the outcome of the disease (**Figure 1**). Additionally, many groups have used biochemical, serological or more recently molecular tools such as multi-locus sequence typing (MLST) or genome sequencing to phylogenetically classify clinical isolate collections, and then correlated this information with patient data as well. This approach has been used extensively to study other pathogens, resulting for example in important insight on the virulence of *Candida albicans* (Rajendran et al., 2016), *Mycobacterium tuberculosis* (Lan et al., 2003) and *Plasmodium falciparum* (Bernabeu et al., 2016). In this paper, we review some of the knowledge we have acquired on human cryptococcosis using these strategies and discuss both existing limitations and what could be done to further progress in this area.

### Virulence and Host-Pathogen Interaction in Humans

The pathogenesis of pulmonary cryptococcosis or cryptococcal meningitis depends on the host immune status and is influenced by several fungal virulence factors (Bicanic and Harrison, 2004). The most studied *Cryptococcus* virulence factors are the polysaccharide capsule (Ngamskulrungroj et al., 2011; Robertson et al., 2014), the ability to produce melanin (Ngamskulrungroj et al., 2011; Renney de Sousa et al., 2020), thermotolerance (Johnston et al., 2016) and the production enzymes such as laccase (Frazão et al., 2020), urease (Shi et al., 2010) and phospholipases (Santangelo et al., 2004).

The capsule was the focus of a study made by Robertson and colleagues with *Cryptococcus* spp. isolates from HIV-infected Ugandan adults as part of a clinical trial (Robertson et al., 2014). They measured the capsule thickness and capsular polysaccharide shedding both *ex vivo*, in the patients’ cerebrospinal fluid (CSF), and *in vitro*, after culture in the laboratory. There was no correlation between the two sets of measurements, an indication that the complex environment inside the human host is not fully reproduced in the laboratory. Interestingly, the authors found that isolates with larger *ex vivo* capsules were found in patients with higher CSF cryptococcal antigen titers, less intense central nervous system (CNS) inflammation and higher intracranial pressure. Beyond the capsule, other morphologic aspects of the *Cryptococcus* spp. cell affect patient outcomes (Fernandes et al., 2018). *C. neoformans* and *C. tetragattii* isolates from Botswana were cultivated *in vitro* and observed microscopically, with multiple morphologies such as giant cells, micro cells and shed capsule fragments. Higher pleomorphism, with a more diverse mix of cell shapes and sizes, was associated with increased risk of death.

The important role of the CNS environment for disease outcome was also highlighted by a second study from the same group (Sabiiti et al., 2014). This study, performed with clinical isolates obtained during five clinical trials in Thailand and South Africa, focused on the fungal interaction with macrophages and on melanin and the enzyme that synthesizes this pigment, laccase. The authors found that some isolates were more efficiently phagocytosed by macrophages than others, and that those with higher *in vitro* uptake by macrophages were isolated from patients with higher CSF fungal burden and higher risk of death. The high-uptake strains were also hypocapsular and had higher laccase activity than the low-uptake strains. Surprisingly, uptake by macrophages correlated positively with laccase activity but not with total melanin produced by each isolate. This observation hinted at melanin-independent activities for laccase, a hypothesis that was proven by *in vitro* exposure of the clinical isolates to CSF. The fluid was toxic to fungi, but strains with increased laccase activity were able to survive better.

Melanin-independent roles for laccase were also central in two recent studies we conducted with Brazilian *Cryptococcus* spp. Isolates. In one of them, we measured the non-lytic exocytosis of a small set of *C. neoformans* isolates (Frazão et al., 2020). This is a process in which macrophages expel *Cryptococcus* spp. cells without harm to either cell (Alvarez and Casadevall, 2006; Ma et al., 2006), and its mechanism is not fully understood. We found that isolates that melanized faster were expelled more frequently. Further experiments showed that what affected the non-lytic exocytosis was actually laccase, not melanin, because pre-melanizing cells did not alter the rates but knocking out laccase did. In another study, at the moment of writing still at the preprint stage (Renney de Sousa et al., 2020), we measured multiple virulence attributes of *C. neoformans* and *C. deuterogattii* isolates, especially the capsule and laccase/melanin. As previously reported (Robertson et al., 2014; Fernandes et al., 2018), we did not observe any correlations between *in vitro* capsule phenotypes and clinical outcomes; we also observed that secreted laccase activity negatively correlates with survival in both cryptococcosis patients and *Galleria mellonella* animal models, in line with previous observations as well (Sabiiti et al., 2014). However, we did observe an important role for melanization rate in survival of both *G. mellonella* and humans, irrespective of laccase secretion. The risk of death was positively associated with how fast each isolate melanized.

The interaction between *C. neoformans* and murine macrophages was the focus of a study made with clinical isolates obtained in a French prospective study (Alanio et al., 2011). Alanio and collaborators observed great variation in the phagocytosis and intracellular proliferation of fungi inside infected macrophages. Isolates that more effectively evaded phagocytosis (low phagocytic index) and had lower intracellular proliferation rates were associated with a higher risk of therapeutic failure, determined as lack of CSF sterilization at 2 weeks. Isolates that were more efficiently phagocytosed but proliferated more efficiently inside macrophages were obtained from patients with a higher risk of death. These findings confirmed that the *Cryptococcus*-macrophage interaction, which in studies with mice and other animal models is crucial for disease outcome, is also important in infected humans. Another study with clinical isolates also found clinical outcomes that parallel those of mouse models (Mukaremera et al., 2019). Using an inhalation infection model, the authors show a strong association between the mortality rates in humans and mice. They have also found that survival in both hosts is dependent on the burden of fungal cells in affected tissues and on each isolate’s growth speed and resistance to stresses.

### Genotypic Diversity

For many years, *C. neoformans* was considered a unique species with four serotypes based on differences in capsule antigenicity. Further characterization of those serotypes revealed epidemiological, biochemical, and genetic differences among them that lead to their subdivision into four varieties: *C. neoformans* var. *grubii* (serotype A), *C. neoformans* var. *gattii* (serotypes B and C) and *C. neoformans* var. *neoformans* (serotype D). Genetics and molecular biology advances during the last two decades resulted in more precise phylogenetic characterization of the different varieties leading first to their separation into two species with different molecular types (*C. neoformans* VNI, VNII, VNB, VNIII and VNIV and *C. gattii* VGI, VGII, VGIII and VGIV) and later in seven different species (Kwon-Chung and Varma, 2006; Lin and Heitman, 2006; Hagen et al., 2015). Species in the *C. neoformans* complex are usually opportunistic, causing mostly meningoencephalitis in immunocompromised individuals. In contrast, those in the *C. gattii* complex are more frequently primary pathogens and are associated with pneumonia in addition to meningoencephalitis (Galanis and MacDougall, 2010).

Most genetic studies with clinical isolates focus on the geographic distribution of the different molecular types, with only a few presenting correlations with clinical data. Most of these are focused on the *C. neoformans* complex, especially the most common VNI (*C. neoformans strictu sensu*) molecular type, the most frequent cause of cryptococcosis worldwide. Wiesner and collaborators found a significant association between the genotype and phenotype of clinical strains of *C. neoformans* VNI and severity of cryptococcal meningitis (Wiesner et al., 2012). Multilocus sequencing typing (MLST) of their isolates resulted in four clonal clusters and three nonredundant evolutionary groups which presented significant differences in patient mortality. The isolates obtained from patients with higher mortality also presented increased capsular polysaccharide shedding and induced a more pronounced Th2 response *ex vivo*. Similarly, other studies have shown that specific subgroups within the *C. neoformans* VNI clade are associated with more or less severe disease:

- A specific sequence type named ST5 is associated with the development of lymphadenopathy and higher blood lymphocyte counts in patients, as well as with worse disability outcomes in people who were cured (Day et al., 2017).
- Among HIV-positive patients from five countries in Asia and Africa, three VNI subclades were found. Individuals infected with isolates from the VNIa-93 subclade, commonly reported in Uganda and Malawi, had lower mortality than those infected with the VNIa-4 and VNIa-5 subclades (Ashton et al., 2019).

As for other clades within the *C. neoformans* complex, two other studies have correlated molecular and clinical data. Using isolates from HIV-positive patients in South Africa, Beale and colleagues found that the VNB lineage was linked to worse patient survival (Beale et al., 2015). They also found an association between the fungal genotype and virulence phenotypes in isolates from the VNII lineage, which had increased laccase activity and *ex vivo* survival on CSF, fungal phenotypes that were previously associated with poorer disease outcomes (Sabiiti et al., 2014). The second report was from the French Cryptococcosis Study Group, who characterized *C. neoformans* isolates from the hybrid AD serotype (Desnos-Ollivier et al., 2015). Their data showed that patients infected with AD hybrids had lower fungal dissemination, less frequent lung involvement and more frequent CSF sterilization after treatment compared to patients infected with either serotype A or D.

Most of what has been learned about human cryptococcosis with this strategy of correlating molecular and patient data has been done with multi-locus sequence typing. The most recent studies, however, have begun to use whole genome sequencing, which is far more robust and results in richer, more detailed data. Gerstein and collaborators have used whole genome sequence typing to characterize a set of clinical isolates (Gerstein et al., 2019). Their work, however, highlights a new tool that can greatly enhance how much information we get from this type of study: genome-wide association studies. The authors identified hundreds of polymorphisms in the genome sequences and correlated those with clinical data, as well as with laboratory information on virulence and host-pathogen interaction. This strategy resulted in 40 *C. neoformans* genes associated with human disease, many of them with unknown functions. Knockout strains were available for 17 of these, and mouse models proved that 35% were indeed less virulent than the wild-type *C. neoformans*. We believe this powerful new approach will result in very important information in the next few years.

## The Way Forward – Multinational Collaboration

The selected studies we reviewed above have brought important insights on *Cryptococcus* spp. virulence and the host response in cryptococcosis. They have, however, important limitations. One of these limitations is intrinsic to the experimental strategy itself: correlation does not imply causation. Experiments like these are thus often followed up with additional experiments to confirm hypotheses raised by the correlations with clinical data. Other important limitations are:

- Most of the papers we reviewed were made with dozens or a few hundred isolates that originated from a single geographic region. The small number of isolates tested means that the studies are powered to discover only strong correlations, whereas the similar origin from all isolates reduces the breadth of the diverse genetic landscape being probed.
- Most often different studies are made with different sets of clinical isolates that were collected by different researchers, hindering reproducibility. It is difficult to distribute and share pathogenic isolates and clinical data with other researchers who could count on existing datasets.
- Microbiology techniques and molecular characterization methodologies vary between research groups, as do strategies used to diagnose and treat the disease. The quality of the clinical data collected also varies a lot, with a tendency for more uniform and complete data when the strains originate from clinical trials in comparison with less precise information gathered retrospectively from patient charts in observational studies.
- The same virulence attributes are measured differently by each research group. For example, Robertson et al. measured the cryptococcal capsule sizes from clinical isolates directly out of the CSF (Robertson et al., 2014), whereas we (Frazão et al., 2020; Renney de Sousa et al., 2020) and others (Fernandes et al., 2018) have measured it in isolates grown *in vitro* in different media.
- Different groups correlate virulence and host-pathogen interaction information with different aspects of the patient outcome. Alanio et al. for example, made correlations with therapeutic efficacy, measured by CSF sterilization after two weeks, and death after three months of diagnosis (Alanio et al., 2011). On the other hand, Robertson et al. (2014) correlated their data with intracranial pressure, fungal clearing and CSF inflammation whereas Sabiiti et al. (Sabiiti et al., 2014) used CSF fungal burden and 10-week survival.

**Figure 2:**
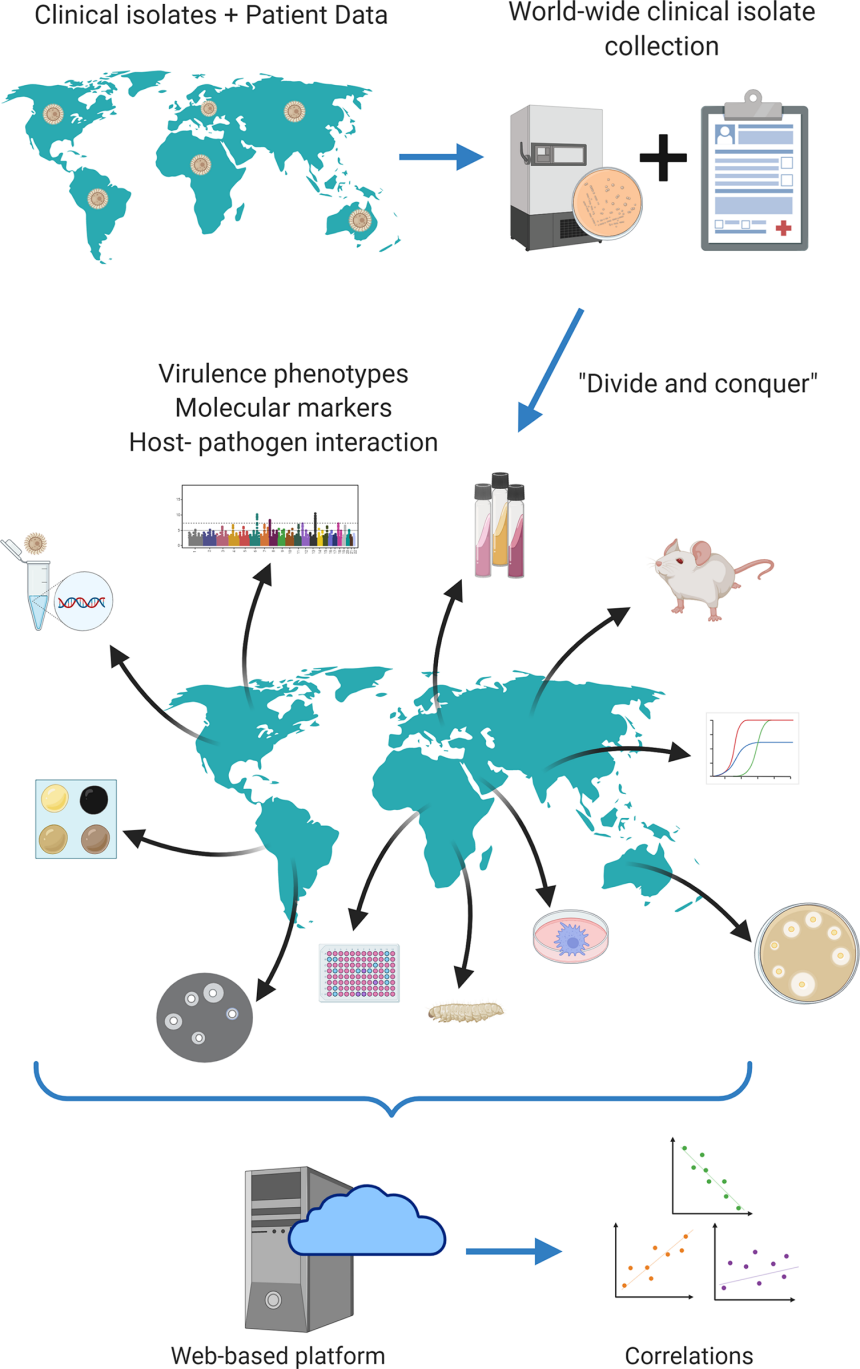
Proposed strategy of translational studies in cryptococcosis as an international collaboration. *Cryptococcus* spp. clinical isolates from all over the world would be gathered in a single biobank; all associated clinical data would equally be gathered in a single open database. Each laboratory would receive all isolates available in the biobank and would be responsible for measuring one or a few virulence factors according to its expertise. All data would finally be combined and analyzed together, increasing statistical power. Figure generated using Biorender.com.

The fact that very important information regarding the pathogenesis of cryptococcosis has been obtained from these studies despite the many limitations means that much more can be understood about the disease with this strategy. Overcoming these limitations, however, would probably require a different approach than what has already been used. Problems with low statistical power and genetic variety can be solved by creating collections with hundreds to thousands of isolates from different regions of the planet (**Figure 2**). Ideally, these isolates would be molecularly characterized with the gold standard (full genome sequencing) and both fungi and associated clinical, molecular, virulence and host-pathogen data would be deposited in publicly available repositories, to allow other researchers to build upon existing knowledge.

This would probably only be possible through worldwide collaborative work between clinicians and basic scientists working on Genomics, Microbiology and Immunology. Such collaboration could also decrease the heterogeneity of patient variables to be collected and the choice of laboratory techniques and data analysis strategies, which could be agreed upon prospectively. This is a complex and laborious proposition, but one that could result in advances that lead to improved outcomes for people dying from cryptococcosis.

## Author Contributions

HS, SF, GO, PA, and AN wrote different sections of the manuscript. AN revised, wrote, and prepared the manuscript. PA and HS prepared the figures. All authors contributed to the article and approved the submitted version.

## Funding

AN was funded by FAP-DF awards 0193.001048/2015-0193.001561/2017 and the CNPq grant 437484/2018-1. PA was funded by FAP-DF grants 193.000.192/2014 and 193.001.003/2015 and CNPq grant 461230/2014-3.

## Conflict of Interest

The authors declare that the research was conducted in the absence of any commercial or financial relationships that could be construed as a potential conflict of interest.
